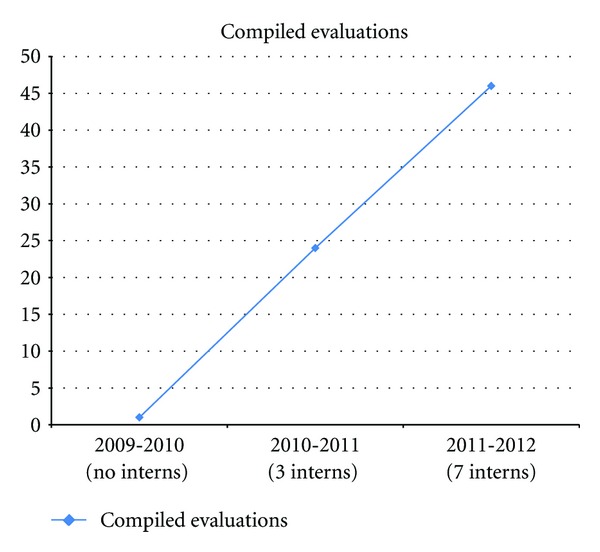# Investing in a Surgical Outcomes Auditing System

**DOI:** 10.1155/2013/671786

**Published:** 2013-01-16

**Authors:** Luis Bermudez, Kristen Trost, Ruben Ayala

**Affiliations:** Research and Outcomes Department, Operation Smile, Inc., Norfolk, VA 23509, USA

## Abstract

*Background*. Humanitarian surgical organizations consider both quantity of patients receiving care and quality of the care provided as a measure of success. However, organizational efficacy is often judged by the percent of resources spent towards direct intervention/surgery, which may discourage investment in an outcomes monitoring system. Operation Smile's established Global Standards of Care mandate minimum patient followup and quality of care. *Purpose*. To determine whether investment of resources in an outcomes monitoring system is necessary and effectively measures success. *Methods*. This paper analyzes the quantity and completeness of data collected over the past four years and compares it against changes in personnel and resources assigned to the program. Operation Smile began investing in multiple resources to obtain the missing data necessary to potentially implement a global Surgical Outcomes Auditing System. Existing personnel resources were restructured to focus on postoperative program implementation, data acquisition and compilation, and training materials used to educate local foundation and international employees. *Results*. An increase in the number of postoperative forms and amount of data being submitted to headquarters occurred. *Conclusions*. Humanitarian surgical organizations would benefit from investment in a surgical outcomes monitoring system in order to demonstrate success and to ameliorate quality of care.

## 1. Introduction

A strong argument can be made that the success of humanitarian surgical organizations must consider both quantity of patients receiving care and the quality of the care provided. However, the efficacy of a nonprofit organization is often judged by asking what percentage of an organization's resources is spent towards direct intervention; in this case, the percentage that goes directly towards providing surgery. Such scrutiny might discourage humanitarian organizations from investing in adequate review of their outcomes, to the detriment of the patients being served. In order for surgical outcomes to be effectively monitored for both patient followup and measuring success, adequate resources need to be allotted towards the establishment of effective systems.

Operation Smile, an international medical nonprofit providing free surgical care for children with clefts, is striving towards measuring its success both by the quantity and quality of care it provides. In 2006, Operation Smile and representatives from its global medical community established the Global Standards of Care, mandating minimum requirements of practice across 14 aspects of care for cleft lip and cleft palate patients. Global Standard 12, titled “Minimum Patient Follow-Up,” states the following:

**Effective post-operative care is essential for a good surgical result and effective planning for further treatment. Post-Operative care requires good documentation and extensive education of parents and clinicians to be effective. Post-Operative care from an Operation Smile organized team should review patients at the following intervals: **

**12.1 One week post-surgery (4–7 days post-op). The goal is to recognize and manage immediate surgical complications. **

**12.2 Six months–1 year. Team evaluation for documenting outcomes of surgeries and planning for future treatment [1].**

This global standard established Operation Smile's commitment to evaluate its success, not just by the number of surgeries provided, but the quality of care. A preliminary Surgical Auditing System was pilot tested in 2008 to determine the effectiveness of evaluating the surgical outcomes of patients [2]. However, In order to implement a large-scale Surgical Outcomes Auditing System, Operation Smile has found that investing in the collection of postoperative data is imperative.

## 2. Measuring Successful Outcomes

 Successful outcomes are often measured by the quantity of services provided to patients. However, the quality of the surgical outcomes should play an equal role in measuring the success of surgery. Good outcomes of primary surgeries reduce the cost spent on secondary revisions as well as ancillary services and procedures. In order to measure success in this manner, a method for evaluating surgical outcomes within a short and a long time frame would be necessary [3].

 The widely accepted goal of cleft care is to return patients to a “normal” life with little to no handicaps associated with cleft lip and/or palate, or the surgical repair. However, measuring this success is often considered complex as it requires the consideration of numerous factors such as velopharyngeal function (speech) palate integrity (fistulas), nasolabial appearance, hearing capabilities, dentoskeletal development, quality of life, and the psychosocial adjustment of the patient. Final assessment of surgical outcomes in the areas of hearing, speech, and skeletal growth require a decent amount of time to pass after surgery. However, nasolabial appearance, facial symmetry, and fistula occurrence have been shown to be effective early measures of surgical outcomes [4–7]. 

## 3. Background

 International humanitarian organizations implement a wide variety of medical mission models. Operation Smile's unique method of utilizing medical missions to build local sustainability has enabled the organization to move from having a mere presence in a country to establishing local foundations and support. There are two main mission methods implemented by Operation Smile. International missions are comprised of team members with at least 50% of volunteers from outside the country in which the mission is occurring and usually lasts 7–10 days, two days of screening, five surgical days, and three days for unpacking and packing of cargo. International missions can take place in both countries that do not have local foundations and countries that do have local foundations. Local missions are comprised of at least 50% local volunteers, volunteers from within the country the mission is occurring. Local missions can last for any length of time, usually between 3 and 10 days and are generally held in countries that have local foundations, which are independently run in-country Operation Smile organization that have the support of Operation Smile International. Occasionally, large unique international missions are implemented for unique projects such as the “World Journey of Smiles,” which took place in 2007. The World Journey of Smiles was a culmination event for the organization's 25th Anniversary in which a large international mission was implemented.

During Operation Smiles' “World Journey of Smiles” in 2007, a Surgical Outcomes Auditing System, using digital photography as media, was developed and pilot tested. Four thousand one hundred patients were operated on in 40 different sites, in 25 different countries over 10 days. During these missions, high-quality images were taken for each patient preoperatively and postoperatively. Postoperative evaluations were held one week after the mission and local foundations were encouraged to schedule six-month and one-year postoperative evaluations for patients. During these postoperative evaluations both standardized images as well as post-operative assessment data collected from PostOperative Exam Forms were collected. This data was then returned to Operation Smile Headquarters where six-month and one-year images were matched with patients' preoperative and immediate postoperative images. Standardized angles of the frontal and basal views as well as images of the hard and soft palates were cropped to protect patients' identities, focusing the reviewer on the surgical area, and to eliminate potential bias of the reviewer [8].

**Figure 1:**
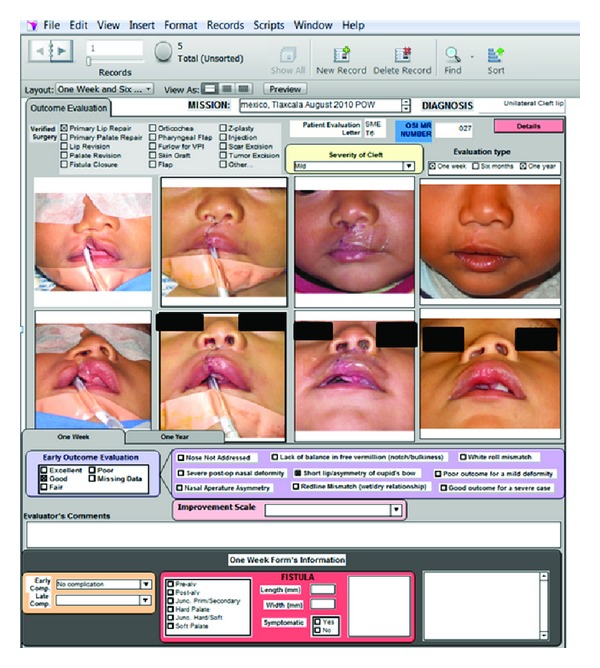


 After compiling this data and deidentifying patient and surgeon information, the evaluations were sent to unbiased members of the International Outcomes Evaluation Council, a group of trained surgical evaluators, and utilizing a qualitative assessment system a surgical evaluation was completed for each procedure provided [2].  Figure 1 shows the final evaluations that were returned to surgeons in a confidential manner. The Regional Medical Officer, who provides medical oversight and leadership for any medical programs within their particular region, and the Medical Director, who is the medical leader for their particular foundation, also received these evaluations to spur further discussion of outcomes.

During the pilot test of the Surgical Outcomes Auditing System, a series of challenges were identified that needed to be overcome. The first and foremost was the need to socialize the concept of auditing the results of surgical procedures and the impact it would have on the mission process. It has taken time and the constant education of medical team members to understand new procedures.

After addressing the challenges associated with the capturing of images, the next challenge to overcome was the quality of the photographic data. At the onset, untrained photographers were being used to collect patient images. With little training in the appropriate image capturing techniques and a thorough understanding of the angles needed, the quality of data obtained was of unusable quality. 

 Once the challenges of acceptance of auditing surgical outcomes and the need for quality trained patient imaging technicians to capture quality standardized images were overcome, the next challenge to overcome was obtaining and analyzing data collected during missions and during postoperative examinations. 

## 4. Method

**Figure 2:**
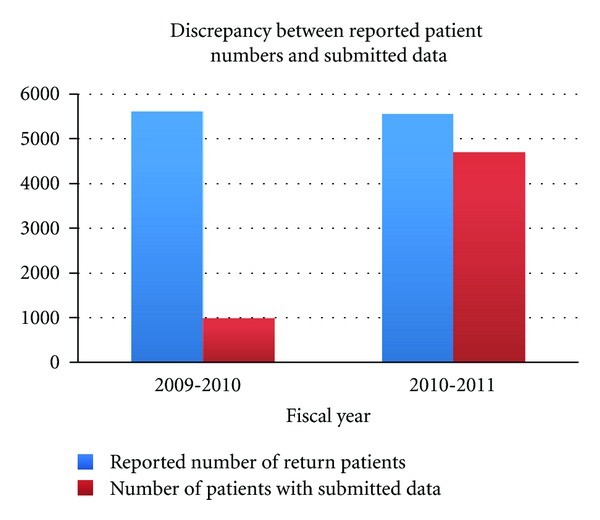


 After the world Journey of Smiles completed in 2007, the postoperative program began to be regularly implemented. Unfortunately, not all of the data from missions were being returned to headquarters.  Figure 2 shows the reported number of patients returning for postoperative examinations in comparison with the data returned to headquarters for these patients.

Recognizing the discrepancy between the number of patients attending post-op and the amount of data received, Operation Smile began investing in multiple resources to obtain the missing data necessary to potentially implement a global Surgical Outcomes Auditing System. Existing personnel resources were restructured to focus on post-op program implementation and data acquisition and compilation. Employees responsible for collecting form data and patient image data went from being members of separate teams within different departments to being on the same team within the same department. This enabled team members to more effectively identify which missions were missing data and initiate the process for obtaining that data.

 As part of this restructuring, personnel began increasing the amount of direct contact with local foundations by phone and e-mail to request the submission of postoperative forms and patient images that had not been submitted to headquarters. Training materials used by these team members to educate local foundation employees and international employees were reorganized to focus more on how to implement properly the postoperative program and to submit data rather than concentrating on how the Surgical Outcomes Auditing System would work. 

Complete data sets, including both patient images and postoperative information, were also not being received from countries in which more local missions were occurring. An Outcome Data Coordinator role was developed to increase the quality and quantity of data return from countries that implemented more locally run missions. Outcomes Data Coordinators became responsible for training local volunteers in the image and form collections process as well as being responsible for collecting, compiling, and submitting this data to headquarters. 

 Foreseeing the need to compile the incoming postoperative data and patient images with minimal employee resources, a data entry intern program was established to deal with data compilation. An internship description was created and posted on the headquarters career website. Applicants with interests in medical, nonprofit, or data entry experience were considered and accepted for positions. 

**Figure 3:**
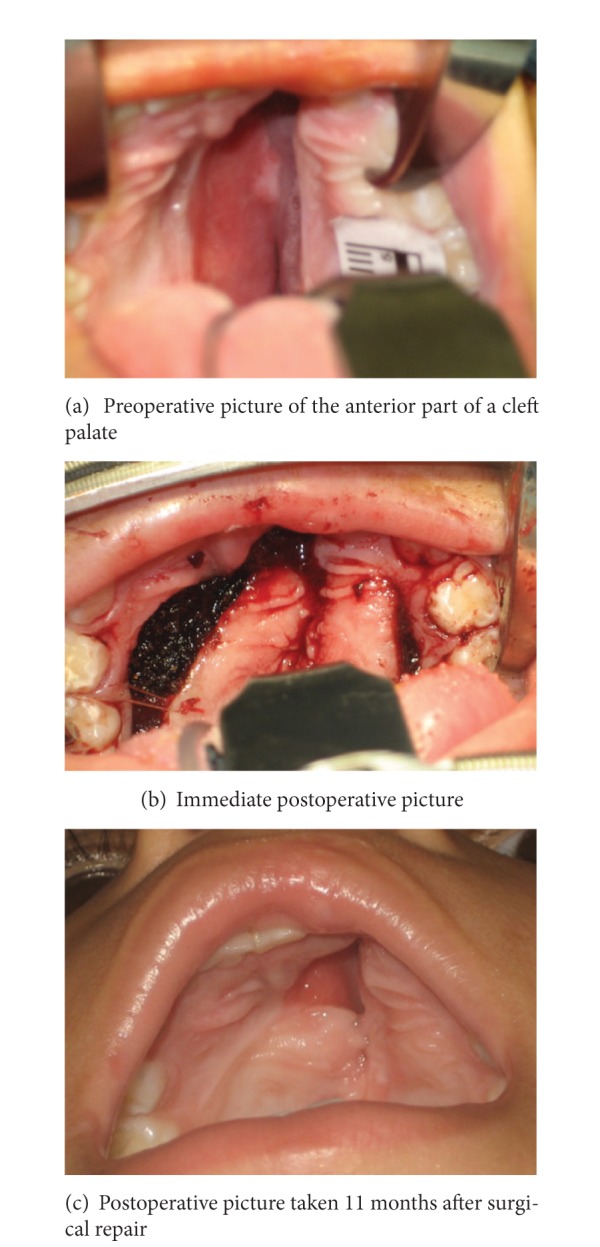
Clinical Case 1.

 To address the increase in one-week postoperative data and the reporting of early complications such as infection and dehiscence, an Early Outcomes Monitoring System was established to identify surgeons with outlying complication rates.  Figure 3 shows an anterior fistula after a cleft palate repair. In the immediate postoperative picture, we can see the presence of a fistula, which was a result of the surgical technique as oral flaps were not designed to achieve complete closure of the anterior palate. Because pictures are taken immediately after surgery, it is often possible to identify the cause of the complication as we can see in Figure 3. These images make it easier to address the complication sooner. 

Recognizing that such complications could be detected earlier, surgical information and postoperative data were combined in an Access database comprised of over 12,000 surgeries with data from the one-week postoperative evaluations of patients operated on by over 400 surgeons. This database enables Operation Smile to monitor the more immediate postoperative complications by surgeon, mission site, and country. While no acceptable complication rate should ever exist, a high variation of complication rates has been reported from other organizations and studies, between 0% and 33% [9–13]. 

 Operation Smile uses these rates as markers to identify surgeons who have outlying complications rates both high and low. When a surgeon is identified as having an outlying complication rate, one that does not fall within the aforementioned range, Operation Smile's Surgical Council receives a report for this surgeon. The report contains information on the cases identified as having complication along with all of the outcomes evaluations on file for the surgeon. This complete report and their surgical cases are then reviewed. The goal is to review and to help in the education of these surgeons, pairing them with mentors and working to enhance their skills.

## 5. Results

**Figure 4:**
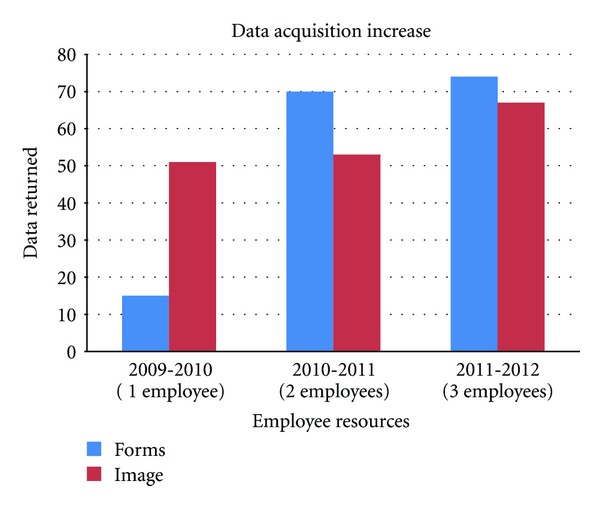


After making direct requests of foundations for specific missing data, an increase in the number of postoperative forms as well as images submitted to headquarters was demonstrated, as seen in Figure 4. 

**Figure 5:**
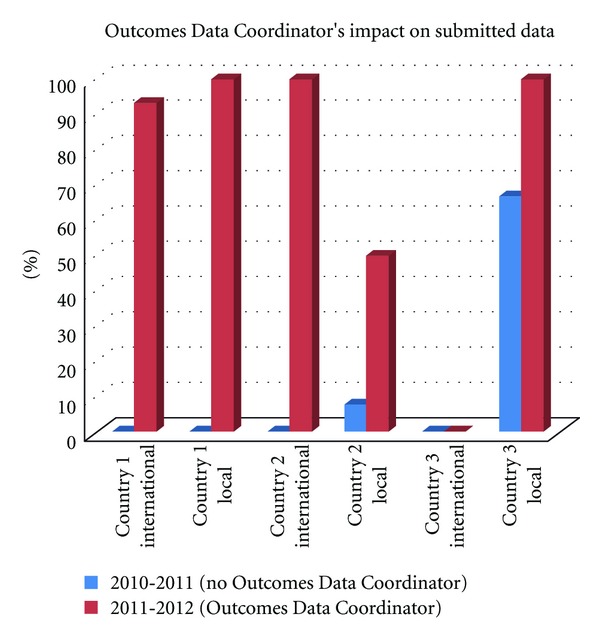


Figure 5, demonstrates the increase in complete data sets submitted to Operation Smile headquarters for both local and international missions by Outcomes Data Coordinators.

As the amount of data being submitted to headquarters increased, the capacity to compile that data was reaching its threshold.  Figure 6 shows that by increasing the number of interns more data could be compiled.

## 6. Discussion 

 Good surgical outcomes are part of Operation Smile's commitment to the patients receiving surgery during its medical missions and at its cleft care centers. In order to meet this commitment and continue to address the surgical needs of children with cleft lip and palate, implementation of a Surgical Outcomes Auditing System has been initiated. Many challenges have played a role in the development and implementation of a global Surgical Outcomes Auditing System, the most impactful of these challenges has been obtaining the necessary data. Recognizing this need, Operation Smile invested in resources to collect and compile the data needed for these evaluations. Through the restructuring and reorganizing of existing personnel, a more cohesive understanding of the specific data needed was gained. This understanding facilitated the direct request of missing postoperative forms and patient images.  Figure 2 shows that the investment in personnel resources resulted in an increase of both postoperative data and patient images being returned to headquarters. Initial data collected was primarily images. Restructuring allowed for more focus on collecting missing data from specific missions. This focus brought an increase in the number of postoperative forms submitted to headquarters. Adding a third employee allowed for more direct contact with local foundations and the ability to request more images as well as postoperative forms, resulting in a further increase in data submitted.

 Another area of focus was on the three countries where high-volume of surgeries were being provided but no data was being received. After the establishment of the Outcomes Data Coordinator position in these three target countries, the percentage of not only data, but complete data sets comprised of patient images and patient information, increased drastically. Countries 2 and 3 increased the amount of international data submitted from less than 50% to 100% and Countries 1 and 3 increased submitted local mission data to 100%.

 Creating a data entry internship program preemptively addressed the challenge of insufficient personnel resources to compile the increase of complete data being submitted to headquarters. As more data arrived due to direct contact and the implementation of the Outcomes Data Coordinator role, more personnel power was needed to compile data into the evaluation templates. Figure 5 demonstrates that as the number of interns doubled so did the amount of compiled evaluations. 

## 7. Conclusion

 Operation Smile's investment in resources significantly increased both the amount of data received and the data compiled from both local and international missions. As local foundations become more and more accustomed to the implementation of the postoperative program as well as the submission of data to headquarters, the more realistic the implementation of a Global Surgical Outcomes Auditing System becomes. However, if Operation Smile is to be successful in obtaining its goal to return patients to a normal life, it cannot limit itself to simply evaluating the visual outcomes of the surgeries they perform.

 Visual outcomes are only some aspects of evaluation that can be used to measure the success of surgical procedures. Speech evaluations can provide further insight into the success of surgeries provided. A perceptual evaluation system has attempted to be implemented in Spanish speaking countries, but the major obstacle is defining what parameters need to be evaluated and how these parameters can be evaluated in order to achieve a good interobserver reliability [14]. Form and functionality is a large portion of returning patients to a “normal life” but it is also very important to determine how well patients are reintegrated into their communities and how they are managing these psychosocial changes.

 As Operation Smile moves forward in the development and implementation of a Global Surgical Auditing System, many other outcomes areas can and should still be measured. With its ever growing global impact through the increasing number of surgeries provided annually, Operation Smile is leading the way in working to ensure that the surgeries they provide are of the best quality.

## Figures and Tables

**Figure 6:**